# Behavioral evidence of a dissociation between voice gender categorization and phoneme categorization using auditory morphed stimuli

**DOI:** 10.3389/fpsyg.2013.01018

**Published:** 2014-01-16

**Authors:** Cyril R. Pernet, Pascal Belin, Anna Jones

**Affiliations:** ^1^Brain Research Imaging Centre, SINAPSE Collaboration, University of EdinburghEdinburgh, UK; ^2^Centre for Cognitive Neuroimaging, Institute of Neuroscience and Psychology, University of GlasgowGlasgow, UK

**Keywords:** voice gender, phonemes, categorical perception, RT, reverse correlation

## Abstract

Both voice gender perception and speech perception rely on neuronal populations located in the peri-sylvian areas. However, whilst functional imaging studies suggest a left vs. right hemisphere and anterior vs. posterior dissociation between voice and speech categorization, psycholinguistic studies on talker variability suggest that these two processes share common mechanisms. In this study, we investigated the categorical perception of voice gender (male vs. female) and phonemes (/pa/ vs. /ta/) using the same stimulus continua generated by morphing. This allowed the investigation of behavioral differences while controlling acoustic characteristics, since the same stimuli were used in both tasks. Despite a higher acoustic dissimilarity between items during the phoneme categorization task (a male and female voice producing the same phonemes) than the gender task (the same person producing 2 phonemes), results showed that speech information is being processed much faster than voice information. In addition, f0 or timbre equalization did not affect RT, which disagrees with the classical psycholinguistic models in which voice information is stripped away or normalized to access phonetic content. Also, despite similar average response (percentages) and perceptual (d') curves, a reverse correlation analysis on acoustic features revealed that only the vowel formant frequencies distinguish stimuli in the gender task, whilst, as expected, the formant frequencies of the consonant distinguished stimuli in the phoneme task. The 2nd set of results thus also disagrees with models postulating that the same acoustic information is used for voice and speech. Altogether these results suggest that voice gender categorization and phoneme categorization are dissociated at an early stage on the basis of different enhanced acoustic features that are diagnostic to the task at hand.

## Introduction

The human voice is what we use to communicate on a daily basis and there is evidence that voices are “special” in that they are processed differently from other stimuli in the brain (Belin et al., 2000, 2004; Petkov et al., 2008). Similarly, there is a large amount of literature showing dedicated processes for speech perception (for example Diehl et al., 2004). In this study we ask if the perceptual processes used to code voice information interact with the ones used to code phonemic information in the speech signal.

Voice perception is the recognition and interpretation of the acoustic structure of the voice. Many factors are likely to be involved in the perception of vocal sounds, both linguistic and paralinguistic. One such factor is the pitch, with the pitch height (the perception of the fundamental frequency f0 of the vocal fold vibration) being its' main characteristic. Timbre (influenced in particular by the distribution of energy across frequencies, as observed in the power spectrum of the sound) is another factor involved in the perception of vocal sounds, and is perceived as the characteristic quality of a sound. Timbre is influenced by the relative strengths of the different frequency components, which in turn are determined by the resonance (Hewlett and Beck, 2004). Although pitch does matter for recognition, is has been suggested that timbre is “integral to recognition mechanisms” (McLachlan and Wilson, 2010) and we can thus expect timbre to be essential to voice recognition as well. Despite fewer studies looking at voice perception neuro-anatomy than speech perception neuro-anatomy, two main brain regions have been identified as supporting voice and voice gender perception. First, voice-selective areas have been demonstrated (Belin et al., 2000; Fecteau et al., 2004, 2005) and are localized bilaterally along the upper bank (middle and anterior) of the Superior Temporal Sulcus (STS) (Belin et al., 2000; Alho et al., 2006), with a predominant role of the right hemisphere (Bestelemeyer et al., 2011). In addition, the categorization of voice gender appears to depend on the right voice selective areas to encode acoustical dissimilarity (Charest et al., 2012). Second, the frontal cortex and in particular the bilateral inferior frontal regions, seem to be important in the encoding of perceived ambiguity and to carry out categorical perception (Charest et al., 2012).

Compared to voice perception, speech perception is better characterized both from a cognitive and neuro-anatomical perspective (Price, 2000; Démonet et al., 2005; Samuel, 2011 for reviews). There is an agreement in the literature that speech sound perception is carried out bilaterally (Binder et al., 1997; Crinion et al., 2003; Scott and Johnsrude, 2003; Saur et al., 2008) with studies showing bilateralization both in brain injured patients (Oscar-Berman et al., 1975; Miceli et al., 1978; Perecman and Kellar, 1981), and healthy volunteers (Sekiyama et al., 2003; Liebenthal et al., 2010). It is however also widely accepted that the main speech-specific regions are left lateralized, with specific involvement of the left lateral superior temporal gyrus (STG) and the mid-posterior STS, lateral and ventral to Heschl's gyrus (Binder et al., 1997; Benson et al., 2001; Dehaene-Lambertz et al., 2005). Of particular interest here is the finding that phoneme perception relies on both the left planum temporal and the posterior STS for spectro-temporal analysis of speech vs. non-speech sounds (Jäncke et al., 2002; Benson et al., 2006; Möttönen et al., 2006) and on the left supra-marginal gyrus for categorical perception (Dehaene-Lambertz et al., 2005).

From a neuroanatomical perspective it thus appears that voice gender categorization and phoneme categorization are dissociated (left vs. right hemisphere dominance, and anterior/mid STS vs. lateral STG and posterior STS). In line with this neuro-functional dissociation, the classic hypothesis in speech perception is that talker (voice specific) information is extracted along with the speech signal first, and is then stripped away to access the phonemic content. This view therefore suggests that voice and speech (as opposed to sound analysis) are processed separately. In contrast to this hypothesis, the effect of talker variability on speech perception has been demonstrated by many. For instance, using a continuous recognition memory procedure, Palmeri et al. (1993) showed that specific voice information is retained in the memory along with item information, and these attributes aid later recognition. Nygaard et al. (1994) showed that learning to identify a talker's voice has an effect on subsequent word recognition performance. Similarly, increased sensitivity to talker-specific information by learning affects the perception of the linguistic properties of speech in isolated words and sentences (Nygaard and Pisoni, 1998). Such results contradict the notion of complete independence and suggest that voice identity perception and speech perception are linked in their perceptual underpinnings. In particular, Remez et al. (1997) show that talker identity can be recognized from sine wave replicas of natural speech that preserved idiosyncratic phonetic variation, thus suggesting that phonetic properties serve to identify both speech and voices.

In an attempt to dissociate these two views, we investigated the pattern of performance of listeners in two orthogonal identification tasks. Using sounds from different continua of “male”-/pa/ to “female”-/ta/ and “male”-/ta/ to “female”-/pa/, subjects categorized stimuli as being either “male”-“female” (gender task) or /pa/-/ta/ (phoneme task). Although other studies have looked at either gender or identity in the context of speech (Lass et al., 1976; Whiteside, 1998; Bachorowski and Owren, 1999; Gelfer and Mikos, 2005), few have tested the two mechanisms simultaneously. Since it has been suggested that voice and phoneme perception rely on similar phonemic properties, but that phoneme categorization must accounts for talker variability (talker normalization), we expected (i) voice gender information to be processed faster than phonemic information (Mullennix and Pisoni, 1990) and (ii) that similar phonetic cues would be used in both tasks. Reaction time (RT) differences between tasks with identical weights of acoustic clues imply a sequence of information processing (i.e., non-dissociated processes) on the basis of shared acoustic information (i.e., the same representations). In contrast, RT differences with different weights of acoustic clues imply parallel and dissociated information processing on the basis of different representations. To further investigate the role of acoustic vs. phonemic features in each task, continua were also equalized in term of pitch height (f0) or timbre (supra-laryngeal filtering). If a normalization process is taking place during the phoneme task, equating sounds in f0 or in some aspect of the timbre should lead to faster RT in those conditions.

In terms of accuracy, and following the results of Pernet and Belin's (2012) who investigated gender categorization in a similar context (but using a single syllable /had/), we expected to observe sigmoid response curves and super-Gaussian perceptual (d′) curves. Both curves are prototypical of categorical responses in 2 alternative forced choice (AFC) designs, although they do not necessarily reflect true categorical spaces (Gerrits and Schouten, 2004). We hypothesized that, in the gender task, significant differences among the original, timbre equalized and f0-equalized sounds would be observed, with altered responses for the timbre equalized sounds. For phonemes, no effect of pitch height or timbre was expected since it is known that phoneme perception in English relies on acoustic clues such as the voice-onset-time (VOT) and formant transitions (Koch et al., 1999).

## Methods

### Participants

Eighteen subjects participated in this study (9 females 35.3 ± 9.2 years old, 9 males 29.1 ± 3.6 years old). Subjects were all healthy volunteers with no known neurological or psychiatric disorder, no uncorrected visual impairment, no uncorrected hearing loss, no speech and language therapy history, no communication impairment and all had English as their first language.

### Paradigm

Subjects were presented with two 2 AFC identification tasks: voice gender (male vs. female) and phoneme (/pa/ vs. /ta/). For each task, there were three conditions (all participants completed all three conditions for both tasks): original sounds, f0-equalized sounds and timbre-equalized sounds. Within each of the three conditions, for each task (gender and phoneme), there were two full continua of morphed sounds: the 1st continuum going from Male-/pa/ to Female-/ta/ and the 2nd continuum going from Male-/ta/ to Female-/pa/. Importantly, the same speakers were used for both continua (the same male pronouncing /pa/ and /ta/ and the same female pronouncing /pa/ and /ta/). In each of the three conditions and for both tasks, each subject heard the following sounds (presented pseudo-randomly) six times each: 100% Male-/pa/; 100% Male-/ta/; 100% Female-/ta/; 100% Female-/pa/; 90% Male-/pa/ and 10% Female-/ta/; 90% Male-/ta/ and 10% Female-/pa/; 80% Male-/pa/ and 20% Female-/ta/; 80% Male-/pa/ and 20% Female-/ta/ etc. for 11 full steps on the morphed continua. Therefore, each participant heard 132 stimuli (2 continua × 11 steps × 6 trials) for each condition they completed. This design allowed us to investigate the effect of the task (i.e., tell if for example the stimulus 80% Male-/pa/ 20% Female-/ta/ was male or female vs. /pa/ or /ta/) while controlling for the general acoustic characteristics of the stimuli since the same stimuli were used in both tasks. However, specific acoustic characteristic could still be identified as the stimuli grouping differed between tasks. In addition, pitch height equalization and timbre equalization (see below “Stimuli”) allowed the specific contribution of these features to the subject responses to be tested. In total, 18 different continua of stimuli were generated from 6 different speakers (3 males and 3 females pronouncing /pa/ and /ta/) and 1 male and 1 female participant carried out all the tasks for each pair of continuum.

Participants carried out the two tasks in six separate sessions (3 phoneme categorization sessions and 3 gender categorization sessions) with an interfering tone discrimination task lasting about 3 min (results not reported here) in the middle of the six sessions. This task was primarily designed to minimize the influence of one task on the other. Tasks and sessions order within tasks were counterbalanced across subjects. Subjects listened to all the sounds via headphones and answered by pressing keys on a keyboard. Key orientation was counterbalanced between participants. Instructions were as follows: “You will hear a series of sounds. You have to decide for each of these sounds whether it sounds more MALE (/PA/) or more FEMALE (/TA/). Here is an example of each of these two categories *(the most extreme sounds from the continuum were played at this point)*. So if the sound you hear is closer to the MALE (/PA/) sound, answer with the key ‘A’ (‘L’); if the sound if closer to the FEMALE (/TA/) sound answer with the key ‘L’ (‘A’). Do you understand?” If the subject did not understand, the examples were played once more and the final two sentences repeated.

### Stimuli

**Figure 1:**
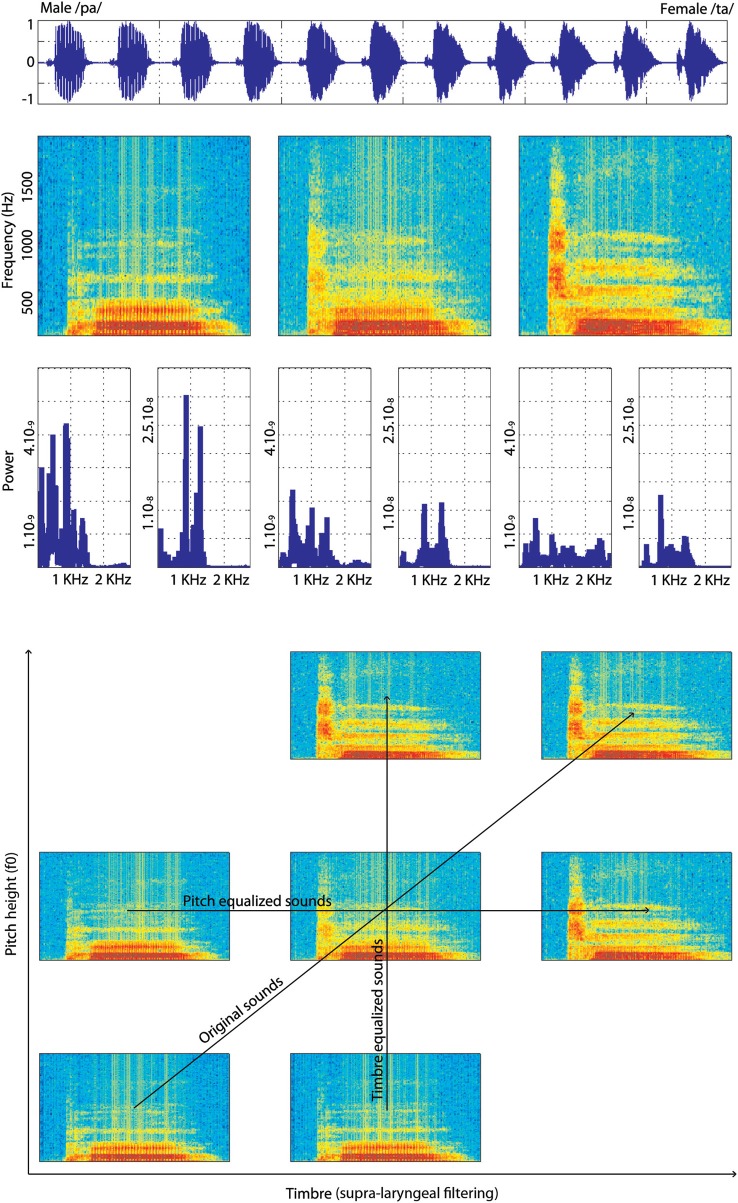
**Illustration of one continuum of male-/pa/ to female-/ta/**. At the **top** is presented a male-/pa/ to female-/ta/ continuum in the time domain (waveforms). Below it, the time-frequency domain (spectrograms with hamming window, sampling at 22,040 Hz) of the 100% male-/pa/ stimulus, 50% male-/pa/, 50% female-/ta/ stimulus and 100% female-/ta/ stimulus from this continuum are shown. The plots below the spectrograms show the power spectra of the consonant and vowel separately for the same stimuli. At the **bottom** of the figure is shown the stimuli “space” with spectrograms of the extreme stimuli (100% male and 100% female) and of the 50% morphed stimulus for each condition: original sounds, f0-equalized or timbre equalized.

Original stimuli were recorded three times each in a sound studio at the Voice Neurocognition Laboratory (http://vnl.psy.gla.ac.uk/). Three males and three females voiced the phonemes /ta/ and /pa/ and stimuli with the clearest separation between the consonant and the vowel (as seen on spectrograms) were selected. Stimuli were then manipulated using STRAIGHT (Kawahara, 2003, 2006) running under Matlab®. STRAIGHT performs an instantaneous pitch-adaptive spectral smoothing in each stimulus for separation of contributions to the voice signal arising from the glottal source vs. supra-laryngeal filtering. The algorithm decomposes a voice stimulus into five parameters: f0, frequency, time, spectro-temporal density and aperiodicity. Stimuli are then synthesized and each parameter can therefore be manipulated and combined across stimuli independently of one another. Here we used time-frequency landmarks to put in correspondence voices, allowing linear morphing. The morphing was based on three temporal (onset of the consonant, onset of the vowel, offset of the vowel) and nine spectral (f0 identified on the consonant and onsets and offsets of the vowel's f0/f1/f2/f3 formants) anchoring points. The morphing was performed from male-/pa/ stimuli to a female-/ta/ stimuli and male-/ta/ stimuli to female-/pa/ stimuli, in nine steps varying by 10% (plus the two original sounds re-synthesized, thereby creating continua containing 11 steps in total). By setting anchoring points on onsets of the consonant and vowels, offset of the vowel and on f0 on the consonant and the vowel, the algorithm could synthesize new stimuli for which the whole sounds were morphs representing a mixture of male-female and /pa/-/ta/. However, by also selecting f1/f2/f3 on the vowel, we forced the algorithm to match these particular spectral points on the vowel. In addition, since the source (f0) and the filter (supra-laryngeal filtering) are dissociated, additional morph continua which were equalized in f0 or in timbre across the stimuli were obtained. For the pitch and timbre equalized continua, the original /pa/ and /ta/ from male and female speakers were first equalized in f0 or timbre and then the morphs were created. Stimuli within each continuum were finally root mean squared normalized (Figure 1).

### Data processing

For each subject, condition, continuum, and morphing step, the 6 scores and RT were collected and cleaned for outliers. S-outliers (deviant from the absolute median distance among all pairs; Rousseeuw and Croux, 1993) were detected from the RTs, and, if any were present, they were removed from the data (both from the scores and RT—8.6% of the data). The mean score (percentage female/ta) and mean RT were then computed. The procedure was iteratively repeated for each stimulus (i.e., 18 subjects, 3 conditions, 2 morphs, 11 steps). From the mean percentages of female/ta responses per continuum, a cumulative Weilbull function was fitted in Matlab® using unconstrained non-linear minimization and the point of subjective equality (PSE: 50% male-female or /pa/-/ta/) was computed. Percentages of correct responses that could not be modeled and/or gave aberrant PSE values were discarded (in total 17.59% of the data). On average, the same amount of data was discarded in each task (13.88% in the gender task vs. 21.29% in the phoneme task, percentile bootstrap confidence interval of the difference [−5.4 3.01]). At this stage, the 2 continua (1. male-/pa/ to female-/ta/ 2. male-/ta/ to female-/pa/) did not differ significantly in terms of percentages or RTs when computed per condition/step (percentile bootstrap on the mean difference with adjustment for multiple comparisons). Averages were thus computed for each condition/step and all following statistical analyses were performed on these averaged scores and RTs cleaned for outlying data points and response curves.

### Data analysis

For all analyses apart from the reverse correlation, 20% trimmed means were used (i.e., computing the mean over 12 participants and removing the three highest and three lowest values). Importantly, analyses on trimmed means give identical results as analyses on means if the data are normally distributed, but they provide a better estimate of the “true” mean for non-normally distributed data. In addition, because significance is obtained using bootstrap procedures, analyses are assumption free. Here trimmed means ensured the data were not biased by inaccurate/slow participants or extremely accurate/fast participants (for comparison of using mean on raw data vs. trimmed mean on cleaned data, see Appendix 1).

Statistical testing within tasks (i.e., original vs. same pitch vs. same timbre) and between tasks (gender vs. phoneme for each condition) was performed using pair-wise comparisons on the 20% trimmed mean difference. For a given comparison, the difference between pairs was computed and 1000 bootstraps obtained (sampling with replacement). The 20% trimmed means were then computed and the percentile CI and *p*-value obtained. Under the null hypothesis of no difference between two conditions, these differences are equally distributed around zero. The *p*-value for an observed difference therefore corresponds to the average number of times the bootstrapped trimmed means were above 0 (or 1 minus this average). It is thus possible to obtain a *p*-value of 0 if all the values are above or below 0. Finally, when multiple pair-wise comparisons were used (e.g., 9 comparisons testing within and between task differences, or 11 comparisons testing between tasks differences along the 11 steps of a continuum), an adjustment for multiple comparisons was applied (Wilcox, 2012).

#### Analysis of percentages of responses

(i) PSEs obtained for each pair of continua were averaged and a percentile bootstrap on trimmed means was computed, testing if the abscissa of the PSE of each condition differed from 6, i.e., the physical middle of the continua. PSEs were also compared with each other (within and between tasks) using pair-wise comparisons. (ii) In addition to this global measure of deviation from the physical middle of the continua, percentages of responses were compared between tasks (gender vs. phoneme) for each of the 11 steps in each condition separately (original sounds, pitch equalized, timbre equalized). (iii) Finally, the rate of change between successive pairs was also tested against 0 (percentile bootstrap on trimmed means) and between tasks. The rate of change was characterized as the perceptual distance (d′) computed between each successive step, exchanging hits/false alarms from one step to the other (Macmillan and Creelman, 2005). While averaged percentages allowed us to investigate absolute differences in categorization performance, using the *d*′-values allowed investigation of perceived distances along the continua.

#### Analysis of reaction times

(i) for each condition and task, the average RTs were computed and pair-wise comparisons were performed within and between tasks. (ii) RTs were compared between tasks (gender vs. phoneme) for each of the 11 steps in each condition separately (original sounds, pitch equalized, timbre equalized). (iii) The rate of change (1st derivative) between successive pairs was also tested between tasks. The rate of change was computed as the average of absolute differences between successive pairs in each continuum. While averaged RTs allowed us to investigate differences in processing time, using the 1st derivative allowed investigation of any significant variations along the continua.

#### Reverse correlations

An analysis of which acoustic features were used by participants to categorize stimuli as “male”-“female” or /pa/-/ta/ was also conducted. Within the gender task, stimuli located below the PSE were categorized as male whilst stimuli located above the PSE were categorized as female. Both categories, however, included /pa/ and /ta/ phonemes and, across participants, different PSE values were obtained and different voice stimuli were used. By comparing the average acoustic properties of perceived male vs. perceived female sounds across participants, it was possible to reveal which acoustic features distinguished male stimuli from female stimuli. Similarly, within the phoneme task, stimuli located below the PSE were categorized as /pa/ whilst stimuli located above the PSE were categorized as /ta/. Both categories, however, included male and female voices and, across participants, different PSE values were obtained and different voice stimuli were used. By comparing the average acoustic properties of perceived /pa/ vs. perceived /ta/ sounds across participants, it was possible to reveal which acoustic features distinguished the two phonemes. Finally, since the same stimuli were used for both tasks, differences in which acoustic properties distinguished male-female from /pa/-/ta/ stimuli should reveal which features were diagnostic to the task at hand.

Using the Praat software (Boersm and Weenink, 2009), the fundamental frequency (mean f0) and Harmonic to Noise Ratio (HNR) of each sound was obtained. In addition, the consonants (/p/ or /t/) and vowel (/a/) were analyzed separately to obtain the mean f1, f2, f3, and f4 formant positions. For each stimulus, the consonant and the vowel were extracted manually [from 0 ms to the onset of the vowel (=consonant) and from the onset of the vowel to the end of the stimulus (=vowel)] and formant values obtained automatically with Praat (search settings from 0 to 5500 Hz, with 25 ms windows and 30 dB dynamic range). The reverse correlation analyses consisted of testing for differences in these sound properties [f0, HNR and formant dispersion (the average frequency difference between consecutive formants, f2/f1, f3/f2, and f4/f3)] for stimuli located above or below the PSE. First, for each subject, each condition and each continuum, the average f0, HNR and formant dispersions were computed separately for stimuli below and above the PSE. Second, a percentile bootstrap on the median differences (Wilcox, 2012) across subjects was computed, thus revealing the acoustic properties that differed among stimuli classified as “male” vs. “female” or /pa/ vs. /ta/. The median was used (rather than the trimmed mean as use previously) because differences in acoustic features were often close to uniformally distributed (see Figure 4). In addition to these comparisons, the average f0, HNR and formant dispersions were obtained for an ideal listener. This ideal listener separated all sounds equally, based on acoustical distances (the PSE was always 6, the acoustic middle of the continua, for all stimuli). For the ideal listener, acoustic properties were thus always averaged and compared for stimuli 1–5 vs. 7–11. In contrast, the PSE differed from participant to participant between 5 and 7, and acoustic properties could be averaged for stimuli 1–4 vs. 6–11 in one participant and for stimuli 1–6 vs. 8–11 in another participant. Comparing the results from the ideal listener to the ones observed in our participant population thus revealed biases in the information used, only if there was a consistent behavior across subjects. The difference between the observed differences in acoustic properties and the differences obtained with the ideal listener were compared using, once again, a percentile bootstrap on the median differences.

## Results

### Percentages of responses

**Figure 2:**
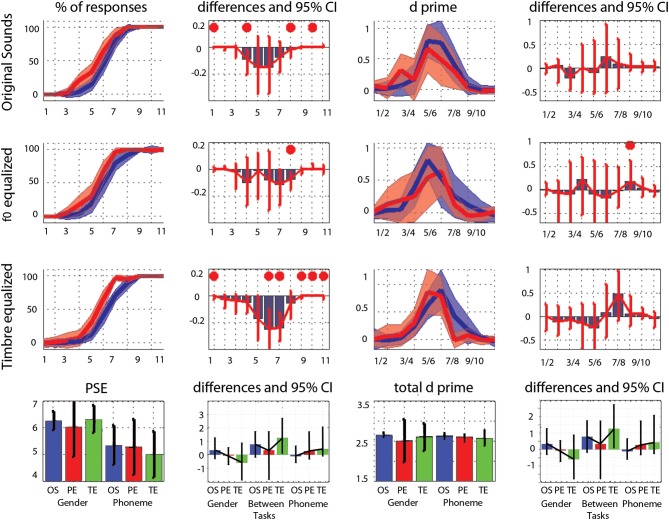
**Trimmed mean responses between tasks and per condition**. For the first three rows, from **left** to **right** are displayed: (i) the 95% CI of response curves in the gender task (blue, percentage of female responses) and in the phoneme task (red, percentage of /ta/ responses), (ii) the differences between tasks in percentage of responses computed for each step (significant differences with adjustment for multiple comparisons marked by a red dot), (iii) the 95% CI of the d' measured between each successive pairs, and (iv) the differences between tasks in d' computed for each step. At the bottom, summary measures are presented. From **left** to **right**: (i) the average PSE, (ii) the within and between tasks differences in PSE (OS: original sounds, PE: f0-equalized sounds, TE: timbre equalized sounds), (iii) the total (cumulative) d', (iv) the within and between tasks differences in total d'. For all graphs, bars represent the adjusted 95% CI.

The average PSE was located at the middle of the physical continuum in the gender task, for all three conditions (original, pitch equalized, and timbre equalized sounds). In the phoneme task, the abscissa was significantly smaller than 6 (biased toward /pa/) for pitch and timbre equalized stimuli (Table 1). Pair-wise comparisons did not show significant difference within tasks (i.e., among conditions) but a significant difference between tasks was observed for the timbre equalized condition (Table 1 and Figure 2). Analyses of percentages of responses for each step separately showed higher ratings in the phoneme task than the gender task for steps 1, 4, 8, and 10 in the original sounds condition, for step 8 in the f0-equalized condition, and steps 1, 6, 7, 9, 10, and 11 in the timbre equalized condition (Figure 2 and Table 2).

**Table 1:** **Trimmed mean PSE with 95% CI (in square brackets) for each task and condition along with the *p*-value associated to the test of difference from 6**.

	**Original sounds**	**Pitch equalized**	**Timbre equalized**
Gender task	6.26 [5.9 6.6]	6.03 [4.9 7.14]	6.31 [5.8 6.83]
	*p* = 0.09	*p* = 0.47	*p* = 0.04
Phoneme task	5.34 [4.6 6.08]	**5.27 [4.24 6.31]**	**5 [4.14 5.85]**
	*p* = 0.01	***p* = 0.003**	***p* = 0**
Difference	0.7 [−0.19 1.7]	0.3 [−1.6 1.8]	**1.2 [0.008 2.75]**
	*p* = 0.04	*p* = 0.5	***p* = 0.004**

*At the bottom of the table are presented the trimmed mean differences between tasks. Significant p-values are marked in bold (alpha adjusted for multiple comparisons)*.

**Table 2:**
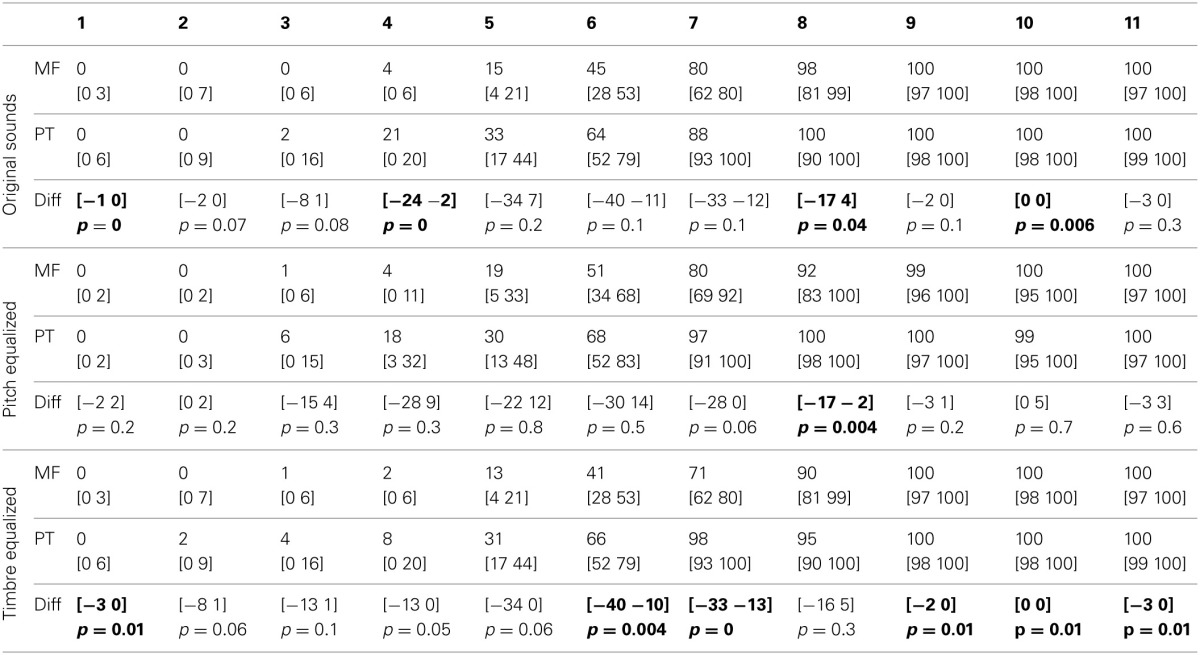
**Trimmed mean percentages and 95% CI of “female” or “ta” responses for each task and condition, and 95% CI and *p*-values of differences between tasks**.

*Significant p-values are marked in bold (alpha adjusted for multiple comparisons)*.

Analysis of the rate of change between successive stimuli revealed, as expected, a significant increase in the perceptual distance for ambiguous stimuli (Figure 2 and Table 3– d′ significantly different from 0). In the gender task, stimulus pair 5/6, 6/7, and 7/8 differed from 0 for the original sounds, stimulus pair 4/5, 5/6, and 6/7 differed from 0 for the f0-equalized sounds, and stimulus pairs 4/5, 5/6, 6/7, and 7/8 differed from 0 for the timbre equalized sounds. In the phoneme task, stimulus pair 5/6, 6/7, and 7/8 differed from 0 for the original sounds, stimulus pairs 5/6 and 6/7 differed from 0 for the f0-equalized sounds, and stimulus pairs 4/5, 5/6, and 6/7 differed from 0 for the timbre equalized sounds. Despite those variations, no significant differences (except pair 8/9 for f0-equalized stimuli—Table 3) between tasks were observed on d′ when testing along the 10 distances, i.e., perceptual distances between consecutive stimuli were equivalent between tasks, leading to similar total d prime (i.e., the cumulative distance from step 1 to 11, Figure 2).

**Table 3:**
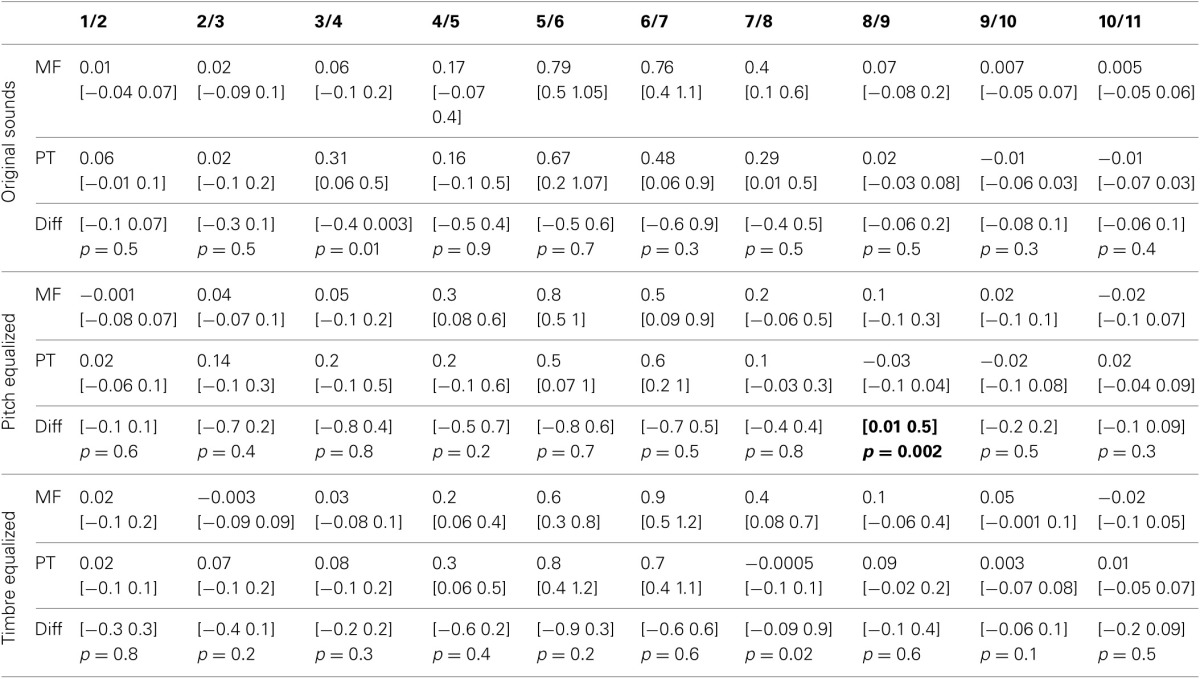
**Trimmed mean *d*′-values and 95% CI for each task and condition, and 95% confidence intervals and *p*-values of differences between tasks**.

*Significant p-values are marked in bold (alpha adjusted for multiple comparisons)*.

### Reaction times

**Figure 3:**
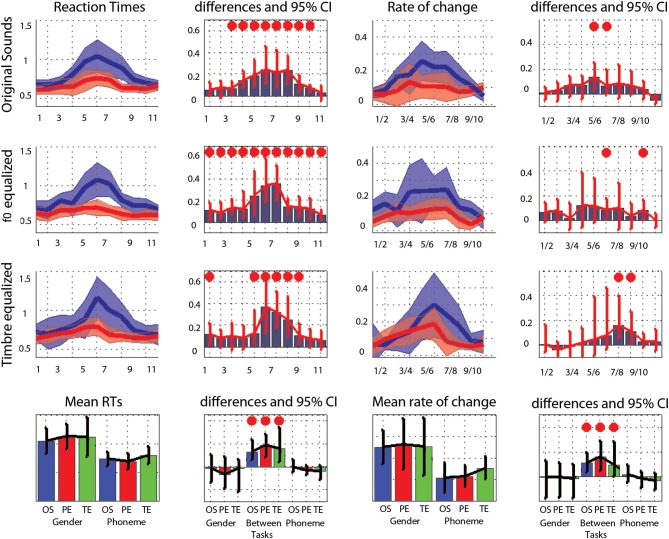
**Trimmed mean reaction times between tasks and per condition**. For the first three rows, from **left** to **right** are displayed: (i) the 95% CI of reaction times curves in the gender task (blue) and the phoneme task (red), (ii) the differences between tasks in RT computed for each step (significant differences with adjustment for multiple comparisons marked by a red dot), (iii) the rate of change in RT measured between each successive pairs (1st derivative) (iv) the differences between tasks in the rate of change computed for each step (significant differences with adjustment for multiple comparisons marked by a red dot). At the bottom, summary measures are presented. From **left** to **right**: (i) the average reaction times, (ii) the within and between tasks differences in average reaction times (OS: original sounds, PE: f0-equalized sounds, TE: timbre equalized sounds), (iii) the average rate of change, and (iv) within and between tasks differences in average rate of change. For all graphs, bars represent the adjusted 95% CI.

Averaged over the 11 steps of each continua, RTs were significantly shorter in the phoneme task than the gender task in each condition (−128 ms [−42 −246 ms] *p* = 0 for original sounds; −180 ms [−66 −269 ms] *p* = 0 for f0-equalized sounds; −172 ms [−81 −381 ms] *p* = 0 for timbre equalized sounds), and no differences were observed within tasks (i.e., between the original sounds, f0-equalized and timbre equalized conditions). When testing for differences between tasks for each condition along the 11 steps, RTs were found to be significantly shorter in the phoneme task (Figure 3) from steps 3–10 with the original sounds (max −241 ms at step 6), for all 11 steps in the pitch equalized condition (max −337 ms at step 7), and for steps 1, 5, 6, 7, 8, and 9 in the timbre equalized condition (max −464 ms at step 6) as shown in Table 4.

**Table 4:**
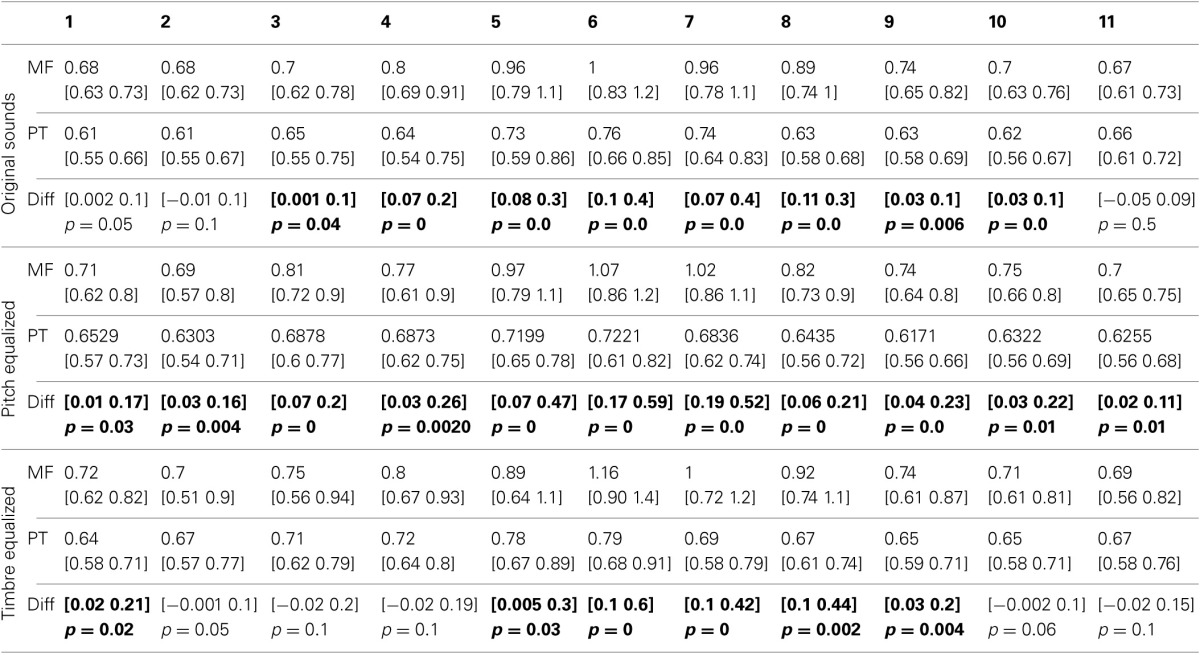
**Trimmed mean RTs and 95% CI for each task and condition, with 95% confidence intervals and *p*-values of differences between tasks**.

*Significant p-values are marked in bold (alpha adjusted for multiple comparisons)*.

The average rate of change along the 11 steps showed significantly larger changes in the gender task than in the phoneme task (0.17 vs. 0.10 ms difference = [0.05 0.22 ms] *p* = 0 for the original sounds; 0.18 vs. 0.10 ms difference = [0.07 0.27 ms] *p* = 0 for the f0-equalized sounds; 0.17 vs. 0.13 ms difference = [0.07 0.37 ms] *p* = 0.002 for the timbre equalized sounds) vs., again, no differences within tasks. Analysis of the rate of change between steps revealed significantly larger changes in the gender task from steps 5 to 6 and steps 6 to 7 and significantly smaller changes from steps 10 to 11 with the original sounds; significantly larger changes from steps 6 to 7 and from steps 9 to 10 with the f0-equalized sounds; and significant larger changes from steps 7 to 8 and from steps 8 to 9 with the timbre equalized sounds (Figure 3 and Table 5).

**Table 5:**
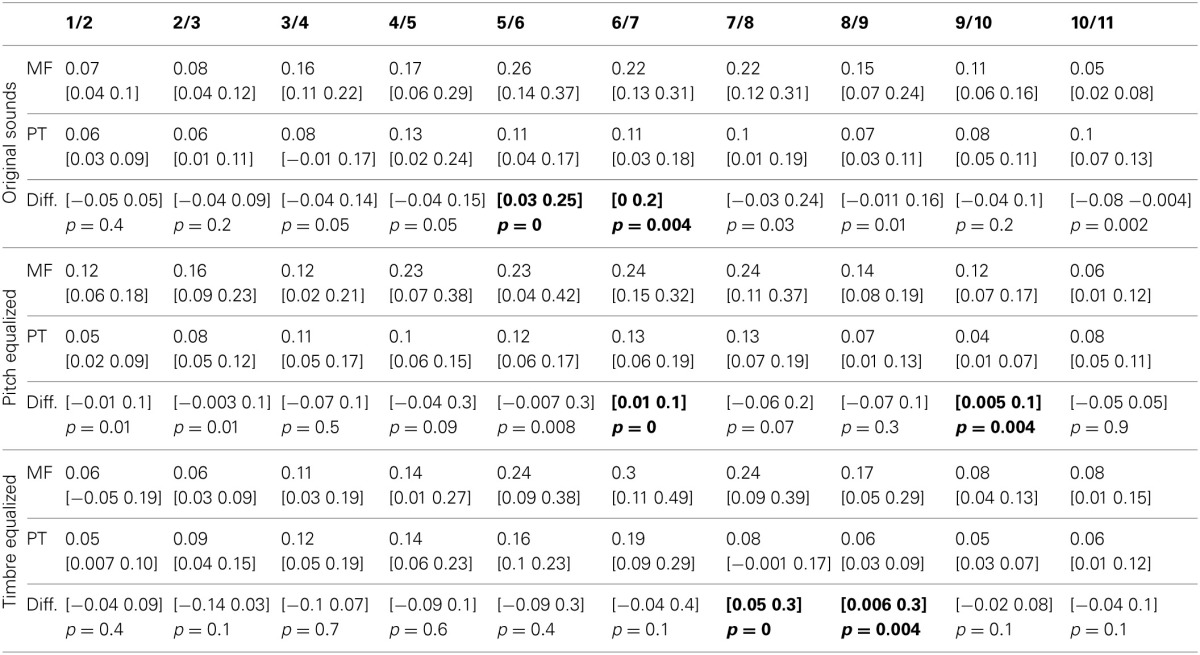
**Trimmed mean of the rate of change in RTs (1st derivative) and 95% CI for each task and condition, with 95% confidence intervals and *p*-values of differences between tasks**.

*Significant p-values are marked in bold (alpha adjusted for multiple comparisons)*.

### Reverse correlations

**Figure 4:**
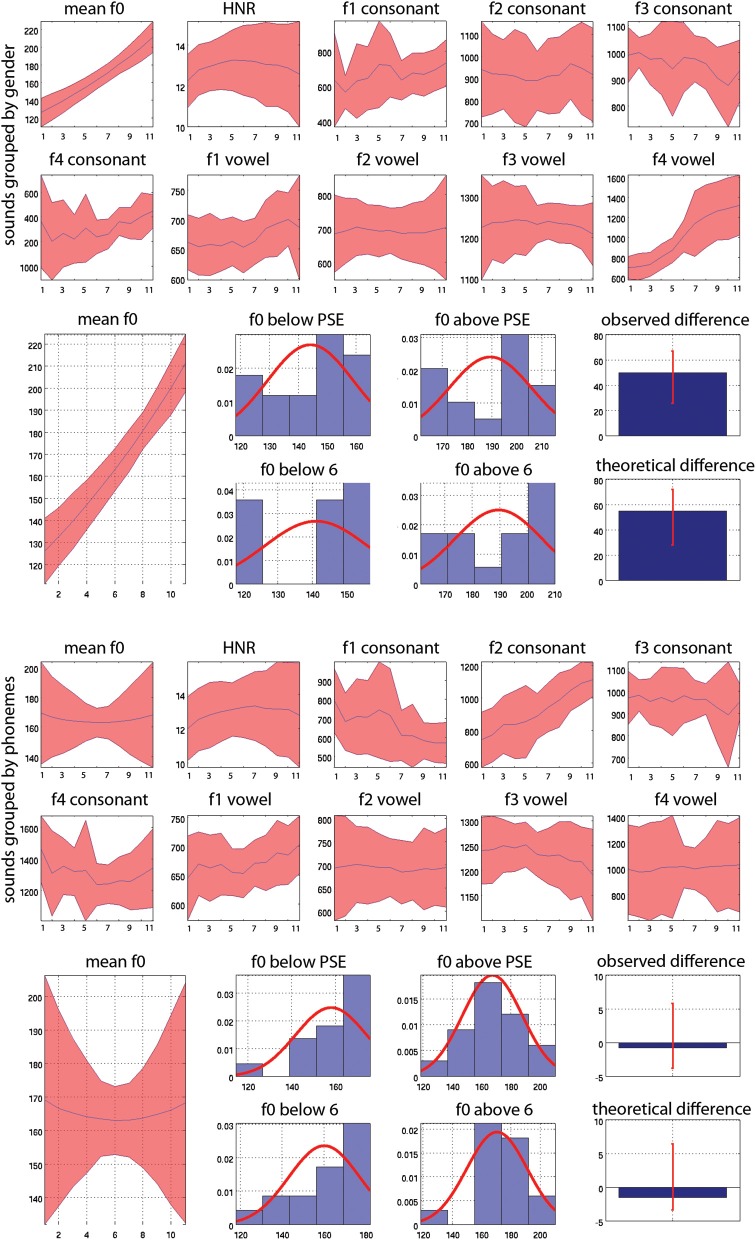
**Mean values and 95% CI of acoustic properties measured on whole sounds (f0 and HNR) and on consonants and vowels separately (f1, f2, f3, f4)**. At the top (1st and 2nd rows) the acoustic properties are averaged according to gender, averaging values across stimuli for each step from step 1 all 100% male stimuli (and therefore averaging male /pa/ and /ta/ together) to step 11, all 100% female stimuli (and therefore averaging male /pa/ and /ta/ together). Below (row 3) is illustrated the reverse correlation for f0: stimuli are separated as below or above the PSE for each subject or as below/above 6 (the actual physical middle) for the ideal listener and then averaged. Histograms show the distribution of f0 values for stimuli classified as males (below PSE or 6) and as females (above PSE or 6) separately. The median differences (bar graphs) are then computed, here showing higher f0 values in “females” than “males.” These differences are next also compared to each other to investigate whether subjects relied more or less on a given acoustic feature than the ideal listener (not showed here). At the bottom (rows 4 and 5) the acoustic properties are averaged according to phoneme, averaging values across stimuli for each step, from step 1, all 100% /pa/ (and therefore averaging /pa/ male and female together) to step 11, all 100% /ta/ stimuli (and therefore averaging /ta/ male and female stimuli). Below this (row 6) is illustrated the reverse correlation for f0: stimuli are separated as below or above the PSE for subjects or above/below 6 (the actual physical middle) for the ideal listener and then averaged. Histograms show the distribution of f0 values for stimuli classified as /pa/ and as /ta/ separately. The median differences (bar graphs) are then computed, showing no differences in f0 here. These differences are also compared to each other to investigate whether subjects relied more or less on a given acoustic feature than the ideal listener (not showed here).

The average acoustic properties measured for the original sounds are displayed Figure 4. As illustrated, ranking stimuli from male to female (gender task—top) or from /pa/ to /ta/ (phoneme task—bottom) gives different results. For instance focusing on the vowel, f0 is higher in the female stimuli (step 11—/pa/ female and /ta/ female stimuli averaged) than in the male stimuli (step 1—/pa/ male and /ta/ male stimuli averaged). In contrast, f0 does not change among the /ta/ stimuli (step 11—male /ta/ and female /ta/ stimuli averaged) and the /pa/ stimuli (step 1—male /pa/ and female /pa/ stimuli averaged). This is explained by the fact that we used two symmetric continua per subject. One continuum was going from male-/pa/ to female-/ta/ whilst the other was going from male-/ta/ to female-/pa/, and was “reversed” in the phoneme task. This therefore cancels acoustic differences such as f0 observed in the gender task. By averaging acoustic properties across stimuli according to the PSE and by task, it was possible to highlight which acoustic features are distinctive between categories (for instance f0 allows to distinguish males from females but not /pa/ from /ta/) and within tasks. Note that this is different from looking at the extremes of the continua and comparing stimuli which would instead only reflect differences from the design. By taking the median difference of the average of stimuli above and below the physical middle (ideal listener) and the PSE (real subjects) we can test if there is a difference between stimuli. It is also important to appreciate that despite supra-laryngeal filtering equalization (i.e., timbre), the vowels from the same and different speakers can have different formant values because the consonant environment influences the formant pattern in vowels (Hillenbrand et al., 2001).

For the voice gender categorization task, comparisons of sound properties for original sounds categorized as “females” had, as expected, a significantly higher fundamental frequency (mean f0) but also and mainly a higher f3–f4 formant dispersion on the vowel than stimuli categorized as “males.” These effects were observed for both the ideal listener and using subjects' categorization performances (Table 6). Comparison of the results from the ideal listener and from subjects' categorization performances show however that a smaller difference on f0 in our participants than expected (f0 difference [−5 −2] *p* = 0, f3–f4 difference [−6 565] *p* = 0.02). For f0-equalized sounds, reverse correlations based on the ideal listener and on subjects' performances show that stimuli categorized as “female” had a significantly higher f3–f4 formant dispersion on the vowel (Table 7), with a smaller difference for the observed than ideal differences (difference [−72 −33] *p* = 0). Finally, for timbre equalized sounds, the reverse correlations on the ideal listener and subjects' performances show that stimuli categorized as “female” had significantly higher fundamental frequency (mean f0), f3–f4 formant dispersion on the consonant and f2–f3 formant dispersion on the vowel. In addition, a significantly higher HNR was also obtained, but only based on subjects' performances (Table 8). Comparisons between ideal and observed results revealed smaller differences on f0 and HNR in our participants than expected (f0 difference [−5 −2] *p* = 0, HNR difference [−0.4 −0.1] *p* = 0; f3–f4 difference for the formant dispersion on the consonant [−9 16] *p* = 0.1 and f2–f3 difference for the formant dispersion on the vowel [−6 13] *p* = 0.15.

**Table 6:**
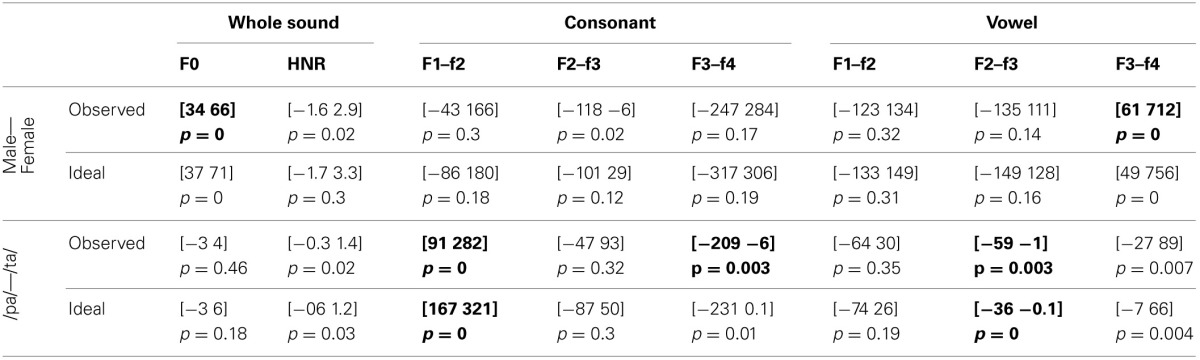
**Reverse correlation results for original sounds**.

*In brackets are presented the 95% CI of median difference between stimuli located above and below the PSE (observed) or above and below 6 (ideal). Significant p-values are marked in bold (alpha adjusted for multiple comparisons)*.

**Table 7:**
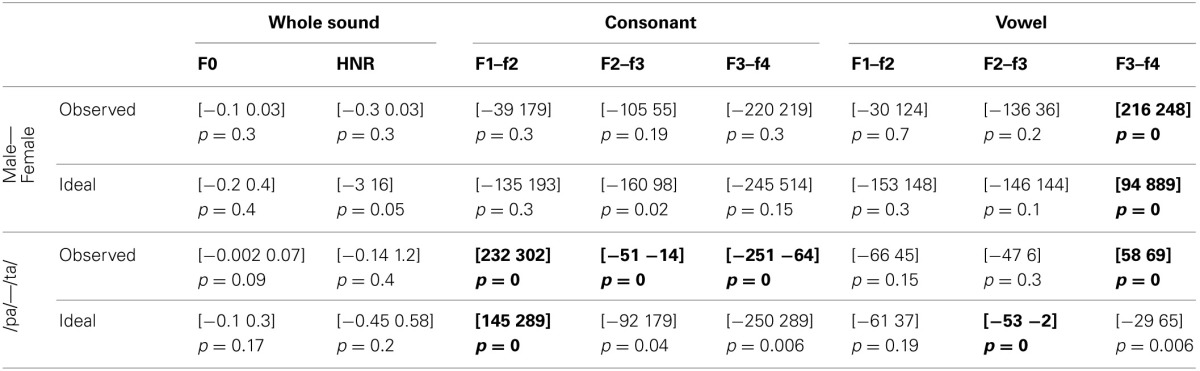
**Reverse correlation results for f0-equalized sounds**.

*In brackets are presented the 95% CI of median difference between stimuli located above and below the PSE (observed) or above and below 6 (ideal). Significant p-values are marked in bold (alpha adjusted for multiple comparisons)*.

**Table 8:**
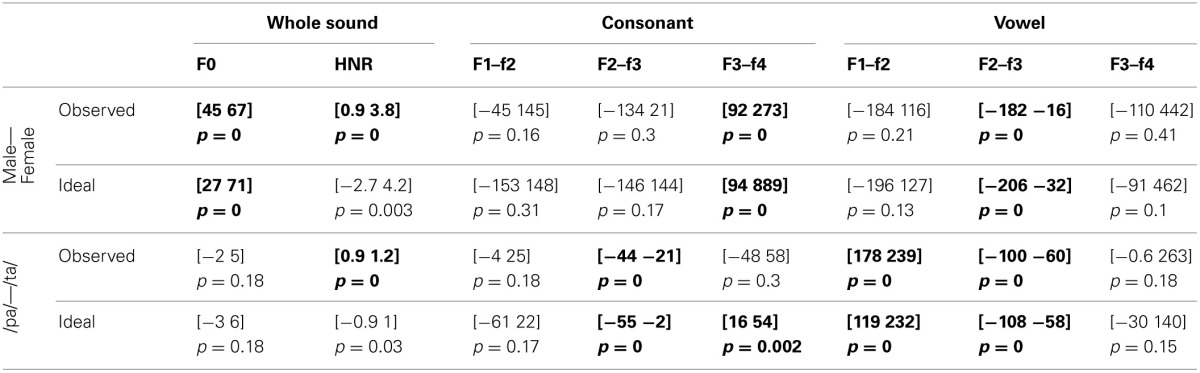
**Reverse correlation results for timbre equalized sounds**.

*In brackets are presented the 95% CI of median difference between stimuli located above and below the PSE (observed) or above and below 6 (ideal). Significant p-values are marked in bold (alpha adjusted for multiple comparisons)*.

For the phoneme categorization task, comparisons of sound properties for original sounds show that stimuli categorized as /pa/ and /ta/ differed mainly on the f1/f2 and f3/f4 formant dispersion on the consonant, but also on the f2/f3 on the vowel. Results from the ideal listener showed significant differences on the f1/f2 formant dispersion on the consonant and f2/f3 on the vowel (Table 6). Comparison of the results from the ideal listener and from subjects' categorization performances showed stronger f1/f2 formant transition of the consonant than expected (f1/f2 consonant difference [−64 −7] *p* = 0; f3/f4 consonant difference [−90 54] *p* = 0.36; f2/f3 vowel difference [−27 15] *p* = 0.04]). For f0-equalized sounds, stimuli categorized as /ta/ by subjects differed from stimuli categorized as /pa/, with higher f1–f2 and lower f2–f3 and f3–f4 dispersions on the consonant (i.e., all formants from the consonants) and higher f3–f4 formant dispersion on the vowel. Comparisons of sound properties based on the ideal listener show differences on the f1–f2 formant dispersions of the consonant and f2–f3 formant dispersions of the vowel (Table 7). Comparisons between observed and ideal results showed no differences (f1/f2 consonant difference [−24 30] *p* = 0.4; f2/f3 consonant difference [−16 5] *p* = 0.4; f3/f4 consonant difference [−11 5] *p* = 0.4; f3/f4 vowel difference [−15 14] *p* = 0.2]). Finally, for timbre equalized sounds, stimuli categorized by subjects as /ta/ vs. /pa/ differed in terms of HNR, f2/f3 formant dispersion on the consonant, and f1/f2, f2/f3 dispersions on the vowel. Comparisons of sound properties based on the ideal listener show differences on the f2/f3 and f3/f4 formant dispersion on the consonant, and f1/f2, f2/f3 dispersions on the vowel (Table 8). Comparisons between observed and ideal results showed a smaller difference for the f2/f3 dispersion on the vowel (consonants: f2/f3 difference [−0.04 0.19] *p* = 0.19, f3/f4 difference [−5 8] *p* = 0.2; vowels: f1/f2 difference [−15 7] *p* = 0.2, f2/f3 difference [−9 −1] *p* = 0).

## Discussion

Categorical responses were observed in all conditions with no *within* category differences seen in perceptual distances compared to sharp *between* category differences (where d′ is different from 0 for ambiguous stimuli). Comparison between tasks revealed a higher rating in the phoneme task than in the gender task (especially in the timbre equalized condition), and faster processing in the phoneme task than in the gender task (the opposite of what was hypothesized). No effect of timbre equalization was observed in the gender task, contrary to what has been previously reported (Pernet and Belin's, 2012). Reverse correlations showed significant differences in vowel formant dispersions when stimuli were categorized as male vs. female, and significant differences in both consonant and vowel formant dispersions when stimuli were categorized as /pa/ vs. /ta/.

### Is voice stripped away from speech?

While we expected voice gender information to be processed faster than phonemic information, because of (i) a higher acoustic similarity between stimuli grouped by talker than grouped by phoneme and (ii) the hypothesized need for talker normalization, we observed the opposite results, i.e., faster RTs in the phoneme task. In addition, if a normalization process was taking place during the phoneme task, equating sounds in f0 or in some aspect of the timbre should have led to faster RTs in those conditions, which was not the case. Together, these results infirm the hypothesis that voice information is stripped away or normalized to access phonemic content.

Taking a purely acoustic view, and following the lawful relationship between sound source mechanics and acoustical structure (e.g., Fletcher and Rossing, 1991), pairs characterized by different sources (i.e., 2 speakers as in the phoneme task) are more dissimilar than pairs characterized by the same source (i.e., the same speaker as in the gender task). This relationship was confirmed by a cross-correlation analysis performed in both the time and spectral domains for the consonant (/p/ and /t/) and the vowel (/a/) (Appendix 2). It should thus be the case that RTs in the phoneme task are longer. One possible explanation for our result is that gender categorization is harder than phoneme categorization, and RT differences simply indexed differences in the difficulty of the tasks. This seems however unlikely since (i) overall subjects performed with high accuracy in both tasks and (ii) if one task would have been more difficult this should have been the phoneme task for which there is more acoustic dissimilarity. Another explanation for our results comes from the design as revealed by the reverse correlation analysis: the phoneme task relies on consonant analysis whilst the gender task relies on vowel analysis, and thus RT differences reflect the fact that phoneme classification starts sooner, i.e., differences in RT reflect differences in the acoustic cues used. If this is true, RTs in the gender task should be delayed by around 40 ms compared to the phoneme task, which corresponds to the time between the end of the consonant (beginning of the phoneme process) and the end of the vowel (beginning of the voice process). However, our data show a 6-fold increase with the original stimuli (+241 ms), an 8-fold increase with f0-equalized stimuli (+337 ms) and up to a 11-fold increase with the timbre equalized sounds (+464 ms). The fact that manipulation of f0 and timbre do change effect sizes between tasks while the consonant to vowel time delay remains constant speaks in favor of a simple interpretation, i.e., gender categorization takes longer than phoneme categorization. Nevertheless, because only those particular phonemes were used (with the consonant always before the vowel), we cannot completely rule out that RTs are explained by the consonant to vowel delay and replications using different phonemes or using vowels only are needed. This does not change however the fact that equating sounds in f0 or in timbre did not change RTs, which should have been the case if a talker normalization process was taking place.

### Attending to consonant vs. vowel

Previous psycholinguistic studies that investigated the links between talker and speech suggest that similar phonemic cues should be used to identify both voices and words (Remez et al., 1997). Results from the reverse correlation analysis however infirmed this hypothesis, showing that the gender task relied mainly on the vowel formant dispersions, and on f0 when available, while, as expected, the phoneme task relied on the consonant formant dispersions. The lack of importance of f0 in phoneme categorization (as shown by the reverse correlation analyses) was an expected outcome since phoneme categorization, in English, has been shown to rely on acoustic cues such as VOT and formant transitions (Koch et al., 1999). The stimuli used here were two single syllables containing voiceless stop consonants (/p/ and /t/) of similar VOT. Analysis of the stimuli using the praat software (Boersm and Weenink, 2009) showed no significant difference of VOT between male-/pa/ and female-/ta/ (mean VOT male-/pa/ 55 ± 14 ms vs. mean VOT female-/ta/ 54 ± 14 ms; *p* = 0.21) or between male-/ta/ and female-/pa/ (mean VOT male-/ta/ 54 ± 14 ms vs. mean VOT female-/pa/ 56 ± 15 ms; *p* = 0.26). The main difference between these two consonants was therefore the place of articulation, perceived as the formant dispersion and this is what we observed in the reverse correlation analysis. However, in contradiction with the hypothesis that the same phonemic cues are used for voice and speech, we observed that only the vowel was important for gender categorization (which was observed in all three conditions). This difference shows that different acoustic features are diagnostic for the task at hand (Schyns, 1998; Schyns et al., 2002) and that therefore gender and phoneme are processed on the basis of different perceptual representations.

### The role of pitch and timbre in gender categorization

No major changes in performances or RTs were elicited by timbre equalization in the gender task, contrary to what was hypothesized. This contrasts with (Pernet and Belin's, 2012) study where such manipulation induced a significant flattening of the response curve in a gender task. One possible explanation for this difference is that the effect previously observed for timbre equalized stimuli was specific to the stimuli at hand, i.e., Pernet and Belin's (2012) used a single morph of average voices compared to the 18 different morphed continua used in this study. The other possibility is that stronger acoustic cues were available in the stimuli used in the current study. In the previous experiment, the morphing was between two identical vowels/consonant syllables (/had/) from an average male to an average female speaker and the morphing was performed on all formants. In the current study, the morphing was between two different consonant/vowels syllables (/pa/-/ta/) from different male to female speakers, with the morphing/mixing of formants applied specifically to the vowel only (see method). The morphing was carried out in this manner because mixing the formants on the consonants would have caused all the stimuli to be perceived as /da/. As a consequence, the timbre equalized stimuli differed on the f3–f4 formant dispersions of the consonant (as showed by the reverse correlation analyses from the ideal listener), a difference which was also significant for the stimuli categorized as male/female by the subjects. Therefore, it seems plausible that the lack of flattening of the response curve was caused by this distinct acoustic feature.

It is already known that gender perception is affected by the size of the larynx and vocal tract (Lass and Davis, 1976; Belin et al., 2004) and that gender is perceived using both pitch (Landis et al., 1982) and timbre (Bachorowski and Owren, 1999). However, because of the pitch overlap in the population between males and females (Titze, 1994), pitch alone can be unreliable for gender categorization (Hanson and Chuang, 1999). Previous studies have argued that pitch height (f0) and formants are the most salient cues to distinguish speaker's sex in the context of vowels (Whiteside, 1998) with a major role of f0 (Gelfer and Mikos, 2005). Because voice gender categorization could be performed accurately using timbre information only (i.e., when f0 is identical across all stimuli) in this experiment, as well as in Pernet and Belin's (2012) where /had/ syllables were used, we can conclude that gender categorization is not solely related to pitch height. In addition, reverse correlation results indicated that formants on the vowel were a major feature in distinguishing male from female stimuli (see also Rendall et al., 2005; Ghazanfar and Rendall, 2008). Together, these results demonstrate a predominant role of timbre when carrying out gender categorization in the context of phonemes, with formants rather than pitch height acting as the major cues. Nevertheless, reverse correlation results also showed that, when available, f0 distinguished male and female stimuli, suggesting that pitch height is encoded and used if it is a present feature and contributes to gender categorization as well.

## Conclusion

On one hand, faster RTs observed in the phoneme task than in the gender task, along with the absence of effect of f0 or timbre equalization, suggest that voice is not stripped away from speech to access phonemic content. On the other hand, stronger weight on the consonant formants in the phoneme task and on the vowel formants in the gender categorization task, suggest that different phonemic cues are used to identify talkers and speech. Although our data challenge results from psycholinguistic studies on talker normalization which suggest either a serial processing (voice 1st, speech next, but see Laing et al., 2012) or a common perceptual underpinning (same weights on acoustic cues), they do fit with functional neuro-anatomical data that show distinct neural substrates for voice gender categorization and phoneme categorization. In accordance with our results showing that voice gender categorization takes longer than phoneme categorization, Charest et al. (2009) showed that the processing of speech sounds differ from human voice sounds (e.g., crying, laughing) as early as 80 ms, while voice selective responses (i.e., voice vs. bird songs and environmental sounds) only differ from 170 ms onward. This result was further supported by Latinus and Taylor (2011) who showed that pitch differences are reflected by early auditory responses (primary cortex response range: 30–60 ms) while gender perception was reflected by later brain responses (from 170 ms onward). Finally, the difference between perceived speech and perceived non-speech using identical synthetic stimuli has also been reported and shown as early as 36 ms, stressing the role of top-down mechanisms during auditory perception (Dehaene-Lambertz et al., 2005).

It has been proposed that long term memory interacts with afferent activations at a very early stage in selecting or enhancing important features (McLachlan and Wilson, 2010). Such a mechanism could explain early differences reported by e.g., Charest et al. (2009) or Dehaene-Lambertz et al. (2005). On the basis of these observations, and McLachlan and Wilson's (2010) idea, we propose that, depending on the task, top-down long term memory interactions create expectations that enhanced formants of the consonant in the phoneme task and f0 and formants of the vowel in the gender task. In turn, these differences in feature enhancement led to RT differences because different parts of the brain are processing those specific features (functional segregation). Phoneme categorization requires finer temporal analysis with short lasting spectral differences being perceived on the consonants, a process that depends more heavily on the left hemisphere (Zatorre and Belin, 2001; Poeppel, 2003; Cohen, 2012). Gender categorization requires finer spectral analysis, a process that depends more strongly on the right hemisphere (Zatorre and Belin, 2001). During the phoneme task, specific phonemic features are enhanced and analyzed in the mid-STS, a region involved in both phoneme categorization (Liebenthal et al., 2010) and voice recognition (Belin et al., 2004). In contrast, during the gender task, specific features are enhanced and analyzed in the right anterior STS (Charest et al., 2012), after speech information have been processed (functional integration).

### Conflict of interest statement

The authors declare that the research was conducted in the absence of any commercial or financial relationships that could be construed as a potential conflict of interest.

## Figures and Tables

**Figure A1:**
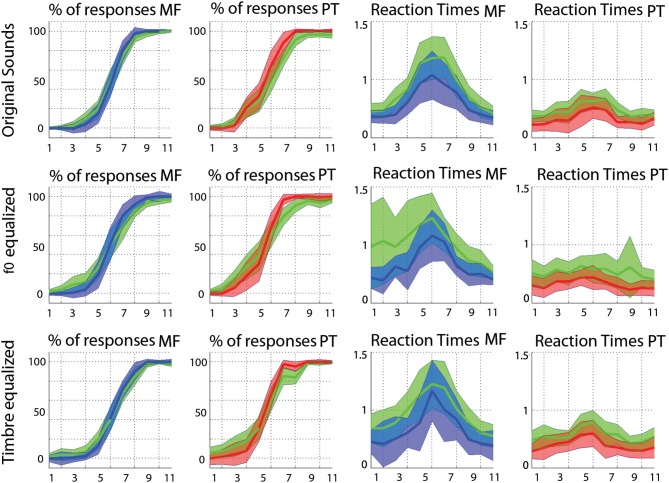
**Comparisons between the “standard” mean computed on raw data with the 95% confidence interval (in green) and the robust alternative: trimmed means on cleaned data with bootstrapped 95% confidence intervals (in blue for the gender task, and in red for phoneme task)**.

**Figure A2:**
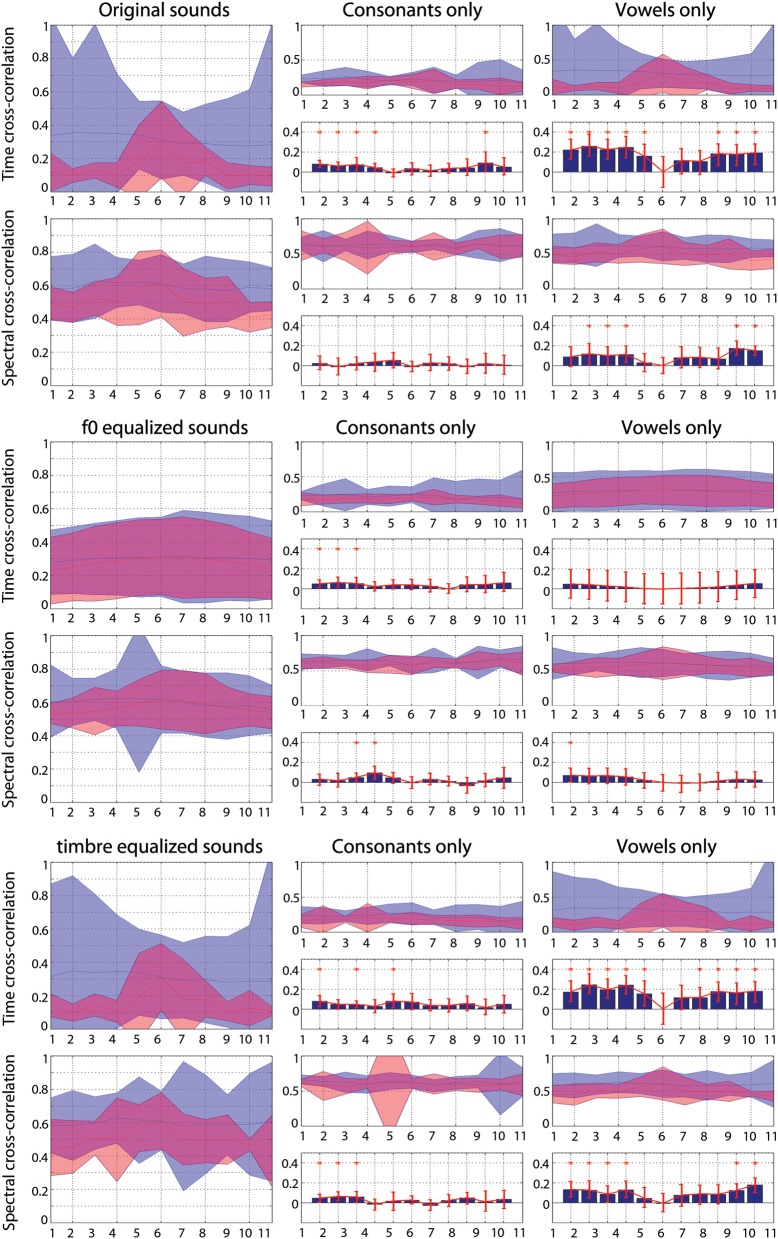
**Cross-correlations between sounds grouped by gender (blue) or phoneme (red)**. Each plain curves represent the 95% confidence intervals of cross-correlation values. High values indicate high similarities. For the consonant and the vowel, pair-wise differences were computed (bar graphs) and significances assessed using a percentile bootstrap (red bars represent 95% confidence intervals—if 0 was not included, the difference is significant).

## Appendix 1

In this article we “cleaned” the data by removing outlier data points based on RTs, allowing the compute the mean performance and RT for each subject. Such data pre-processing is routinely performed by many and is indeed recommended to remove observations that are inconsistent with the remainder of the data (Barnett and Lewis, 1994). They are many relevant techniques to do so, and we used here the S-outlier detection method (median of absolute deviations—Rousseeuw and Croux, 1993) which has a high sensitivity [see e.g., appendix in Pernet et al. (2013)]. Once the mean performances and RTs were obtained, the statistical analysis was performed across subjects using trimmed means. Trimming simply removes the lowest and highest values, and the *p*-value comes from estimating the null hypothesis via bootstrapping. Importantly, although trimmed means reflect the data after the removal of the extreme values, the *t*-tests we carried out account for the total number of subjects using Winsorized variances. Using trimmed means gives identical results as standard means if data are normally distributed, such as results can be interpreted the same way as with means. Data are however almost never normally distributed (e.g., Miceri, 1989) and standard statistics then seriously lack of power. In these cases the mean is a poor estimator of the population average and trimmed means have been shown to reflect better the true underlying average (Wilcox, 2012).

As shown in Figure A1, mean scores and RTs on raw data and trimmed mean scores and RTs on “cleaned” data were quite similar, indicating that data were close to normally distributed. However, it is also apparent that mean response curves are flatter than trimmed mean ones and mean RTs are all longer than trimmed mean ones. Using means on raw data also lead to larger confidence intervals, sometime because of a single data point in one subject, which illustrates that using trimmed means offers a more reliable alternative than means. Finally, and importantly for our results, the relationships between conditions (e.g., faster RTs for phonemes than voices and no effect of f0 and timbre equalization) is unchanged between methods.

## Appendix 2

For each continua, cross-correlations were computed in the time and in the frequency domains (power spectrum computed using an FFT at 1024 Hz and 50% overlapping hamming windows) following the grouping subjects had to perform in each task. For the gender task, correlations were computed between the consonants male-/p/ and male-/t/ or female-/p/ and female-/t/ and between vowels from the same “speaker” (e.g., between the 100% male-/a/ from the stimulus /pa/ and the 100% male-/a/ from the stimulus /ta/). Despite the fact that for the vowel the same speaker is used, some differences are expected because the preceding consonant changes the vowels' formants (Hillenbrand et al., 2001). For the phoneme task, correlations were computed between the consonants male-/p/ and female-/p/ or male-/t/ and female-/t/ and vowels from the same “phoneme” (e.g., between the 100% male-/a/ from the stimulus /pa/ and the 100% female-/a/ from the stimulus /pa/). The cross-correlation values obtained by grouping stimuli according to gender or phoneme were next compared pair-wise using a percentile bootstrap on the mean differences.

Results Figure A2) show that overall the consonants and vowels taken from the same speakers were more similar (higher correlation values) than the consonants and vowels taken from the same phonemes. In the spectral domain, this effect was attenuated although still present depending on the condition.
